# Evaluating a European knowledge hub on climate change in agriculture: Are we building a better connected community?

**DOI:** 10.1007/s11192-016-2064-5

**Published:** 2016-07-15

**Authors:** Eli Rudinow Saetnan, Richard Philip Kipling

**Affiliations:** Institute for Biological, Rural and Environmental Sciences, Aberystwyth University, Aberystwyth, SY23 3DD UK

**Keywords:** Agriculture, Climate change, Interdisciplinary collaboration, Co-authorship networks, EU research policy, Collaborative funding initiatives, Knowledge hub

## Abstract

In order to maintain food security and sustainability of production under climate change, interdisciplinary and international collaboration in research is essential. In the EU, knowledge hubs are important funding instruments for the development of an interconnected European Research Area. Here, network analysis was used to assess whether the pilot knowledge hub MACSUR has affected interdisciplinary collaboration, using co-authorship of peer reviewed articles as a measure of collaboration. The broad community of all authors identified as active in the field of agriculture and climate change was increasingly well connected over the period studied. Between knowledge hub members, changes in network parameters suggest an increase in collaborative interaction beyond that expected due to network growth, and greater than that found in the broader community. Given that interdisciplinary networks often take several years to have an impact on research outputs, these changes within the relatively new MACSUR community provide evidence that the knowledge hub structure has been effective in stimulating collaboration. However, analysis showed that knowledge hub partners were initially well-connected, suggesting that the initiative may have gathered together researchers with particular resources or inclinations towards collaborative working. Long term, consistent funding and ongoing reflection to improve networking structures may be necessary to sustain the early positive signs from MACSUR, to extend its success to a wider community of researchers, or to repeat it in less connected fields of science. Tackling complex challenges such as climate change will require research structures that can effectively support and utilise the diversity of talents beyond the already well-connected core of scientists at major research institutes. But network research shows that this core, well-connected group are vital brokers in achieving wider integration.

## Introduction

The complex societal challenges of climate change and sustainability are now key components of both national and international science strategies and are reflected in national and international funding mechanisms (The Royal Society 2011). It has been widely recognised that tackling such global challenges will require collaboration across disciplines and between nations, to build research capacity, advance knowledge and generate potential solutions (Siedlok and Hibbert 2014; The Royal Society 2011). Climate change is occurring against a backdrop of large scale and complex socio-economic change, including global population increases and dietary changes towards livestock products in the developing world (Thornton 2010; Tilman and Clark 2014). In this context, the agricultural sector is required to create and maintain sustainable production systems capable of ensuring food security in the long term while at the same time addressing the dual challenges of mitigating and adapting to a changing climate (Beddington 2011; Lipper et al. 2014). Internationally, a wide range of initiatives are being developed to address these formidable challenges through coordination and collaboration between countries in research, policy and finance (Lipper et al. 2014). The Joint Programming Initiatives (JPI) of the European Commission are coordinating a European regional research agenda to increase the value of national research programmes (European Commission 2015). Within this framework the JPI on Agriculture, Food Security and Climate Change (FACCE-JPI) (http://www.faccejpi.com) has implemented the “knowledge hub” concept as a new instrument to facilitate and drive collaborative scientific research, in order to develop the coherence of purpose required to efficiently tackle urgent global and multi-sectorial problems such as climate change (Holzinger et al. 2012; Soussana et al. 2012). Here, we aim to evaluate the potential of knowledge hubs to build community cohesion and hence facilitate interdisciplinary collaboration, by comparing the structure of the scientific community before and during the active period of MACSUR (Modelling European Agriculture with Climate Change for Food Security), the first FACCE-JPI Knowledge Hub.

MACSUR (http://macsur.eu) is a pilot knowledge hub created by FACCE-JPI in 2012. It brings together 70 institutes across 18 countries, including modellers and empirical researchers with expertise in grasslands, crops, livestock, farms, and agricultural economics and a particular interest in addressing climate change impacts within these themes (MACSUR 2015). The overarching challenge for MACSUR is to build European capacity in the development, use and interpretation of models to perform risk assessments of the impacts of climate change and adaptation strategies on European agriculture. This is achieved through activities organized around three themes relating to crop, livestock (including permanent grasslands and farms) and trade modelling. In addition, there are cross-cutting activities for integrating knowledge across disciplines. Hence, a major task for MACSUR is to increase collaboration across both disciplinary and national boundaries. The knowledge hub is also expected to engage with a range of stakeholders and governmental policymakers, and in this respect could be described as a ‘multiple helix’ network (Klenk and Hickey 2013) although in this paper the focus is on the role of the project in stimulating inter-disciplinary collaborations between researchers.

### Establishing international collaborations

The scientific community has seen a steady increase in international collaboration over the past century (Adams 2013; Lariviere et al. 2015; Sonnenwald 2007). International collaboration has been shown to enhance the impact of research by increasing the likelihood of publication and increasing the visibility of published work (Katz and Martin 1997; Uddin et al. 2013) with internationally co-authored papers having a higher citation rate (Adams 2013; Glänzel et al. 1999) or higher quality than those produced by colleagues within nations (Barjak and Robinson 2007). In addition, collaborations between researchers with diverse backgrounds and expertise can be particularly beneficial for the generation of novel insights and discoveries (Katz and Martin 1997; Siedlok and Hibbert 2014). This is of particular importance, given the recognition that novel, innovative solutions are required in addition to incremental changes in order to tackle current challenges facing agricultural systems (Martin et al. 2013).

Informal communication appears to be an important aspect in building partnerships; the desire for more social interactions and intellectual stimulation have been identified as motivations for collaboration (van Rijnsoever and Hessels 2011) with Siedlok and Hibbert (2014) highlighting both the pull of interdisciplinary novelty and the push of disciplinary limitations as drivers to work across disciplines. Consistent with these ideas of socially-motivated interaction, collaboration appears to be more likely when potential collaborators are closer either geographically (Hoekman et al. 2010; Katz 1994; Luukkonen et al. 1992) or socially (Katz and Martin 1997). Ensuring a good match of interests and complementarity in skills and personal traits between potential co-workers, are reported as the most important factors determining the level of collaboration between scientists (Hara et al. 2003). The challenge is becoming aware of, and getting to know, potential collaborators. In some research areas, bringing together scientists in a networking activity such as a large conference, has been shown to increase the cohesion and connectedness of the research community (Tomassini and Luthi 2007). Knowledge hubs and other networking instruments may serve a similar function, with the engagement of partners in networking activities facilitating and driving wider and deeper collaborations between community members.

Despite the described advantages of international and interdisciplinary collaboration, building such groups is not a trivial matter. Although it is well established that there is a significant correlation between productivity and collaboration (Lee 2005), scientists have been shown to preferentially continue existing collaborations rather than forming new links (Abbasi et al. 2012). A range of barriers to inter-disciplinary collaboration have been identified, including reward systems aligned to work within disciplines, perceived trade-offs between inter-disciplinary collaboration and career advancement through specialisation, academic prejudice against inter-disciplinary research and differences between disciplinary norms, practices and language (Siedlok and Hibbert 2014). A lack of high quality inter-disciplinary publication platforms may also hinder inter-disciplinary progress in some fields (Brandt et al. 2013), although several well respected interdisciplinary journals are now established in the areas of climate change, environment and agriculture. These factors may help to explain why external incentives for collaboration are only weakly correlated with collaboration frequency (Hara et al. 2003).

The European Union has played an important role in promoting international scientific collaboration and integration in science and technology through its funding frameworks (Glänzel et al. 1999). Within some research fields, a high level of international collaboration has been established among EU states for more than 20 years (Glänzel et al. 1999) and continues to grow (Hoekman et al. 2010). Europe is a heterogeneous region in terms of infrastructure, scientific expertise and historical trajectories, creating potential for co-learning across a number of dimensions. At the same time, geographical and language barriers and an unequal distribution of funding and resources have been factors in determining patterns of collaboration across the region. These challenges remain, although a gradual convergence toward a more integrated European science community appears to be reducing difficulties relating to co-publication across national borders (Hoekman et al. 2010).

The discussion above indicates that initiatives aimed at building inter-disciplinary cohesion can have benefits both for the research community as a whole and for the individual scientists involved. However, the costs to scientists of engagement in inter-disciplinary collaboration mean that effective support and sufficient funding must underpin inter-disciplinary initiatives (Siedlok and Hibbert 2014) if their potential is to be realised. The channelling of resources to such initiatives is only likely to occur if their benefits can be clearly evaluated. The aim of this paper is to evaluate the success of the knowledge hub funding mechanism as a strategy for increasing interdisciplinary and international research collaboration, by studying the structures of collaboration networks over time.

### Visualizing and quantifying research collaborations

Scientific collaboration can be seen as a self-organizing system where co-authorships represent links in a communication network between scientists (Newman 2004; Wagner and Leydesdorff 2005). Network analysis methods can then be used to discover the operating dynamics of this system (Wagner and Leydesdorff 2005). It is very difficult to define the boundaries of research collaboration (Katz and Martin 1997), hence choosing a metric to measure the outcomes of such activities is also challenging. The term ‘collaboration’ itself can refer to a wide range of activities, from general discussion and advice to active participation in data collection or analysis and from substantial to negligible levels of engagement, all of which can be encompassed by eventual co-authorship (Katz and Martin 1997). Although data on co-authorships is easily accessible and verifiable, making it a relatively inexpensive and practical method to use for measuring collaborative interactions (Katz and Martin 1997; Milojevic 2010) the approach is by no means perfect. There can be many reasons why a collaborative effort does not lead to a joint publication, or why co-authorship does not reflect actual collaborative research (Katz and Martin 1997; Melin and Persson 1996). However, co-authorship should at least be positively correlated with collaboration (Glänzel and Schubert 2004). Co-authorship on published papers has been used to investigate aspects of scientific collaboration in many different fields, from mathematics (Brunson et al. 2013; Grossman 2002) to social science (Moody 2004) and engineering (Abbasi et al. 2011). Co-authorship networks are therefore widely recognised as reasonable indicators of scientific collaboration, and provide an objective measure of the effectiveness of structures such as knowledge hubs to increase collaborative research.

Previous analyses of co-authorship networks have found big differences between research fields in terms of their network structure and connectedness, suggesting differences in sociological or organizational collaboration patterns (Belter and Seidel 2013; Newman 2004), though it is unclear what those differences are or why they occur (Newman 2004). Communities of collaborators can be understood as cohesive and well-connected when the majority of authors are connected to each other either directly or indirectly, and the average distance between authors is short (Newman 2004). Such communities can be seen as “small world” networks, centralized around a few well-connected authors (Abbasi et al. 2011) who have the potential to act as brokers of information between collaborators. Most paths through the network connecting one author to another go through these well-connected authors (Newman 2004). In contrast, a community with little evidence of interdisciplinary collaboration produces a highly modular or disconnected network separated into small cliques, with little sharing of information between them.

Much previous work has focused on describing community networks within well-defined disciplinary fields (e.g. mathematics: Brunson et al. 2013; Industrial Ecology: Kim and Perez 2015). Describing co-authorship networks for interdisciplinary fields is more challenging than applying the approach within disciplines, as the boundaries of the focus community are not so clear. This paper attempts to describe the community of scientists represented by the inter-disciplinary knowledge hub MACSUR. The aim of the analysis was to investigate how well integrated the MACSUR community was before the establishment of the knowledge hub, and whether any improvement in community cohesion or levels of collaboration could be detected during its active period. In addition to overall community network dynamics, the position of MACSUR members within the community of Climate Change and Agriculture researchers, and whether that position changed as a consequence of participation in the Knowledge Hub, was also investigated.

## Methods

### Community definition and data collection

In order to identify the community of researchers collaborating in subject areas related to MACSUR, data on co-authorship was extracted from research articles published in journals indexed by the Web of Science (WoS) database, limited to papers published in the period 2008–2012 (before the establishment of MACSUR) and 2012–2014 (during the active period of MACSUR). Although Web of Science is a comprehensive database, and the search generated a very large sample, there are some limitations to relying on this resource alone. Web of Science has a clear bias towards English language journals, with local or domestic journals and journals in other languages under-represented (Hoekman et al. 2010). However, the MACSUR project operates at the level of international collaboration across Europe, the outputs from which are more likely to appear in international than local journals, and as a result the searches conducted are considered sufficient. Web of Science is also biased towards natural science journals, which may have meant that our search missed some potentially relevant economics journals. However, journals relating to agricultural economics, which are most likely to be relevant for this community, are well represented within this database.

Specific search terms were selected, to cover each theme in the knowledge hub (Table 1). The term “model” was not included, as this search proved too restrictive and missed many publications by MACSUR members. Only articles for which at least one author had a European affiliation address were selected for inclusion in the analysis. Results from the three searches were combined in a single database, and duplicate papers removed before further analysis. The analyses described below were carried out for two groups; (1) using all identified papers (‘whole community’ analyses) and, (2) using only those papers including authorship by members of MACSUR (see details below).

**Table 1:** Search terms used to identify publications in topics related to MACSUR, along with the number of papers recovered for each search

Theme	Search terms	Records identified
Crop	Crop Climate change	2145
Livestock	Grassland or livestock or ruminant Climate change	1472
Trade	Econom* or trade Agriculture* Climate change	941

Search terms combined with “AND” operator within each theme. Boolean operators (*) used to capture all relevant variations of a term

### Author disambiguation

The method of author disambiguation can have a big influence on network analysis results (Klosik et al. 2014). Here, an un-restrictive method of disambiguation was used; authors whose surname and first name or surname and all initials were the same were assumed to be the same person and their records merged. This level of author disambiguation has been suggested to result in around 5 % error rate (Newman 2001). The database was further simplified by removing authors who only appear in the whole database once, as these are likely to be transitory in the particular research field.

### Author co-occurrence matrix and network analysis

Collaborations are rarely continued indefinitely; rather they are dependent on current sources of funding and research interests. New authors are continually added to the potential pool of collaborators while others retire or disappear from the academic community. Therefore, the active network is limited to the real or possible collaborations at any given time (Tomassini and Luthi 2007). The focus of this study was on how the active network of ongoing collaborations changed over time, capturing the dynamic nature of the interactions. Previous work has suggested that the active network of scientific collaboration has a sliding time frame of 3–4 years, with longer timeframes likely confounded by the inclusion of collaborations which are no longer active (Tomassini and Luthi 2007). A sliding time-window of 3 years was therefore chosen for this analysis. Co-authorship links added to the network for each time step will then represent either the establishment of a new collaboration or the re-establishment of a former collaboration which has lain dormant for a period of time. The first time window was 2008–2010, well before the start of the MACSUR knowledge hub. The final two time windows, 2011–2013 and 2012–2014, encompass the start and first full active phase of MACSUR. For each time window, the database of authors and papers was re-formatted into a co-occurrence matrix, where a co-occurrence in this instance is the co-authorship of a paper. Based on these matrices, network objects were created for further analysis. Within these networks, individual authors are nodes, and the edges between them indicate connection by co-authorship. Each network can be analysed by calculating a number of parameters, including centrality, clustering coefficient, modularity and the size of the largest component. These parameters are defined as follows:

*Network diameter* and *mean distance*, are measured as the longest possible path and mean shortest path length between two authors (Albert and Barabási 2002). Mean distance is expected to decrease as the network grows, with a lower network diameter being indicative of a more tight knit community with a greater number of co-authors within the same number of edges (Bettencourt et al. 2009).

*Author centrality* includes measures of how well connected an author is within the network. The number of co-authors an individual has during the period under analysis is recorded as *degree centrality*; a count of the number of direct links from a given node (Freeman 1979). *Betweenness centrality* is a measure of how frequently a given node appears along the shortest paths between all other pairs of nodes in the network. A high betweenness centrality indicates an author with the potential to broker and control the flow of information between other nodes in the network (Freeman 1979).

*Network centrality* is a measure of the compactness of the network structure, accounting for the variance in degree or betweenness centrality (number of co-authorship links) between individual authors in the network. This parameter gives an index of the level to which the network is hierarchical with key authors connecting most other authors in the network (Freeman 1979). The value ranges from 0 (i.e. all authors have the same degree or betweenness centrality) to 1 (i.e. one author connects the entire network).

*Network clustering coefficient* measures the proportion of possible links which are actually realised in the network and so is a measure of the extent to which authors in the network tend to group together (Albert and Barabási 2002; Cainelli et al. 2014). The value ranges from 0 (i.e. neighbours of any author are not connected to another neighbour of the same author) to 1 (i.e. an author’s neighbours are also neighbours of each other).

*Network modularity* is a measure of community divisions within the network. Here, a random walk optimization method was used to detect communities within the network. Modularity is then defined as the fraction of co-authorship links within communities minus the expected value of that fraction if the edges are randomized. A high modularity score suggests clear community divisions within the network, with the majority of co-authorships within communities and few co-authorships between communities within the network (Newman 2012).

*Network components* are defined as groups of co-authors which are linked to each other, but do not have links to authors outside the group. If all authors were linked either directly or indirectly to all other authors in the network, the network would consist of only one component.

*Network densification* is a measure of the relationship between the number of nodes and the number of edges as the network grows. This relationship can be described in terms of a simple scale relationship with a scaling exponent *α* (Bettencourt et al. 2009). Research areas with a high degree of shared concepts or practices are expected to show *α* > 1.

In addition to network level parameters, the average degree centrality and betweenness centrality of individual authors was calculated for each time period.

All analyses were conducted in *R* version 2.14.1. Network parameters were calculated using package *iGraph* (Csardi and Nepusz 2006). Modularity and largest components were identified using a random-walk based algorithm (“walktrap community”). Network parameters were compared with a random network with the same number of authors and co-authorship links using the Erdös-Renyi model algorithm in *iGraph*. Bootstrapped (*R* = 1000) 95 % confidence intervals for the random model estimates were created, and observed network parameters considered significant if outside this interval. Degree and betweenness centralities of authors were analysed with a two-way ANOVA with MACSUR membership and time as independent variables.

## Results

The number of papers and authors identified as part of this interdisciplinary research field continued to grow throughout the study period. Though the structure of the whole community network changed little, it remained significantly more clustered and modular than expected by random modelling (Table 2). Centrality of the network remained low throughout the study period, though significantly higher than would be expected in a random network (Table 2).

**Table 2:** Network parameters for the entire network of authors identified in the literature search

Year	2008–2010	2009–2011	2010–2012	2011–2013	2012–2014
Authors	1439	1838	2107	2437	2515
Edges	5102	7184	8406	10,718	13,298
Network diameter	14 (7.14–7.18)	15 (7.02–7.05)	16 (7.00–7.03)	18 (6.68–6.74)	14 (6.01–6.02)
Mean distance	5.27 (3.93–3.94)	5.53 (3.87–3.88)	5.58 (3.91–3.92)	5.50 (3.82–3.83)	5.01 (3.58–3.59)
Network degree centrality	0.09 (0.01 ± 0.00)	0.07 (0.01 ± 0.00	0.07 (0.01 ± 0.00)	0.06 (0.00 ± 0.00)	0.07 (0.01 ± 0.00)
Network betweenness centrality	0.09 (0.01 ± 0.00)	0.12 (0.01 ± 0.00)	0.11 (0.01 ± 0.00)	0.08 (0.00 ± 0.00)	0.08 (0.00 ± 0.00)
Network clustering coefficient	0.62 (0.00 ± 0.00)	0.65 (0.00 ± 0.00)	0.62 (0.00 ± 0.00)	0.62 (0.00 ± 0.00)	0.58 (0.00 ± 0.00)
Network modularity	0.85 (0.27 ± 0.00)	0.85 (0.25 ± 0.00)	0.85 (0.25 ± 0.00)	0.81 (0.23 ± 0.00)	0.76 (0.20 ± 0.00)
Network components	298	326	346	356	367
Largest component	689	993	1240	1589	1597
Largest component %	47.9	54.0	58.9	65.2	63.5

Network structure is compared with a simulated Erdös–Renyi random network (values in parentheses) based on an equivalent number of authors and co-authorship links. Random model estimates are bootstrapped (*R* = 1000) means and 95 % confidence intervals, and observed network parameters are considered significant if they fall outside this range

**Fig. 1:**
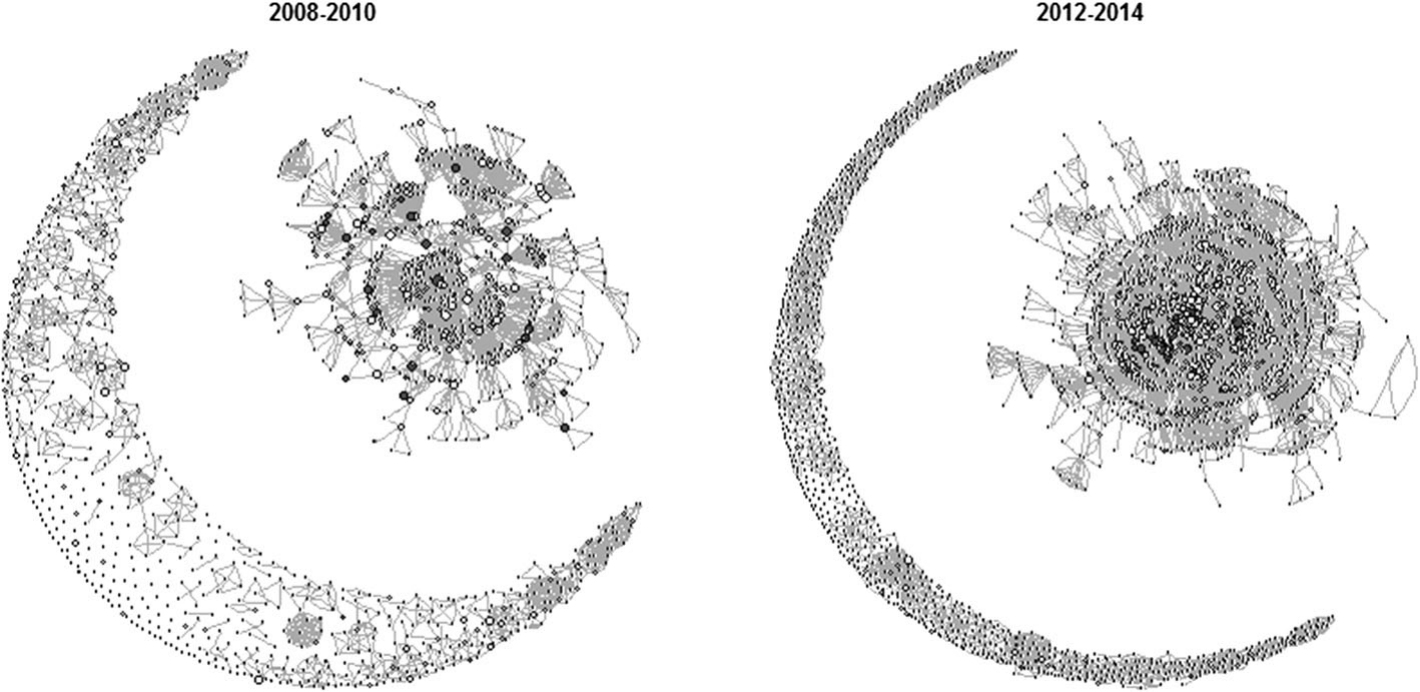
A network graph representing the whole community in the first period (2008–2010) and the final period (2012–2014) of the analysis. Each *node* represents an author, with lines connecting nodes representing co-authorship links. *Nodes* representing MACSUR members are highlighted in *black*

The whole community network structure (Fig. 1) was large and diverse, showing a small increase in cohesiveness by the end of the study period. This appearance was reflected in a small decrease in clustering coefficient and modularity, a small decrease in mean distance, and a small increase in the largest component as a proportion of the whole network (Table 2). The network also showed a growing densification over the study period (*α* = 1.58). However, the growth of the largest component was highly correlated with the growth in the total number of authors in the network (*R* ^2^ = 0.99, *p* < 0.01) suggesting that these changes in network structure were not indicative of a significant change in community dynamics but rather a consequence of increasing community size.

**Fig. 2:**
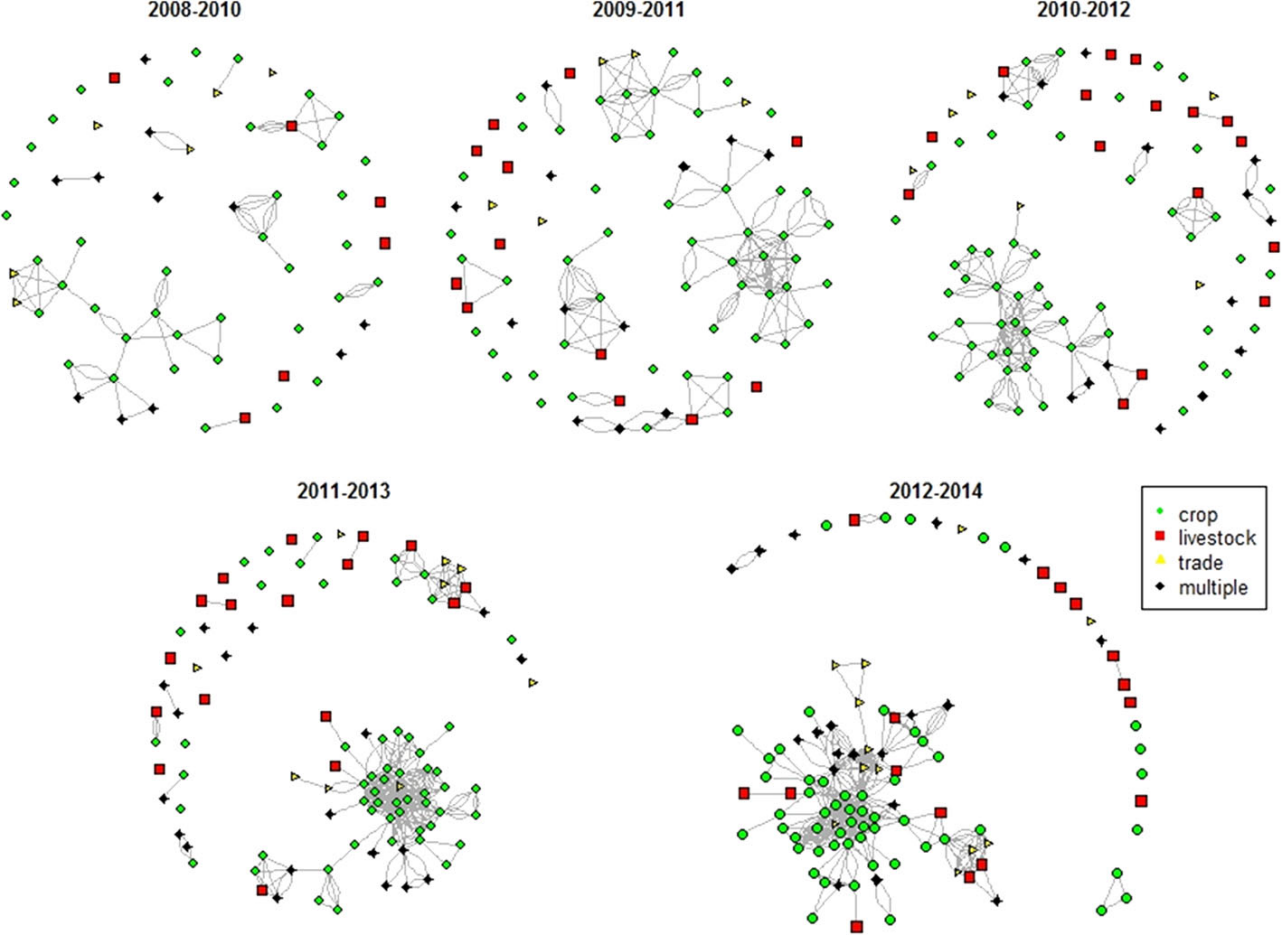
Development of the MACSUR network community before (*top row*) and during (*bottom row*) the active phase of MACSUR knowledge hub. Each *node* represents an individual author, where membership in MACSUR theme groups is indicated by different *colour* and *shape*. (Color figure online)

 The network made up only of MACSUR members, unlike the whole network, showed a clear increase in cohesion during the period studied (Fig. 2). The largest component made up an ever increasing proportion of the network (Table 3), growing more rapidly than the number of authors in the network, with the two only weakly correlated (*R* ^2^ = 0.68, *p* = 0.05). Modularity also decreased considerably during the study period (Table 3) and network densification was far greater than that for the whole network (*α* = 3.42), further suggesting a considerable increase in community cohesion. Mean distance remained relatively low and unchanged during the study period (Table 3) suggesting that, from the start, the community was already more closely knit than expected (Table 3).

**Table 3:** Network parameters for the network made up of only members of the MACSUR knowledge hub

Year	2008–2010	2009–2011	2010–2012	2011–2013	2012–2014
Authors	61	82	90	101	104
Edges	60	140	158	325	401
Network diameter	5 (11.43–11.67)	4 (7.75–7.84)	6 (7.69–7.78)	7 (5.05–5.09)	8 (4.35–4.41)
Mean distance	2.97 (4.83–4.91)	2.15 (3.58–3.59)	2.78 (3.59–3.61)	2.97 (2.66–2.67)	3.26 (2.47–2.48)
Network degree centrality	0.08 (0.07 ± 0.00)	0.19 (0.06 ± 0.00)	0.20 (0.06 ± 0.00)	0.43 (0.07 ± 0.00)	0.39 (0.07 ± 0.00)
Network betweenness centrality	0.06 (0.04 ± 0.00)	0.03 (0.11 ± 0.00)	0.07 (0.10 ± 0.00)	0.11 (0.05 ± 0.00)	0.13 (0.04 ± 0.00)
Network clustering coefficient	0.61 (0.07 ± 0.00)	0.67 (0.04 ± 0.00)	0.60 (0.04 ± 0.00)	0.66 (0.06 ± 0.00)	0.62 (0.07 ± 0.00)
Network modularity	0.81 (0.28 ± 0.00)	0.70 (0.46 ± 0.00)	0.64 (0.45 ± 0.00)	0.38 (0.31 ± 0.00)	0.54 (0.28 ± 0.00)
Network components	31	34	42	31	24
Largest component	19	23	36	54	76
Largest component %	31.1	28.0	40.0	53.5	73.1

Network structure is compared with a simulated Erdös–Renyi random network based on an equivalent number of authors and co-authorship links. Random model estimates are bootstrapped (*R* = 1000) 95 % confidence intervals, and observed network parameters are considered significant if they fall outside this range

**Fig. 3:**
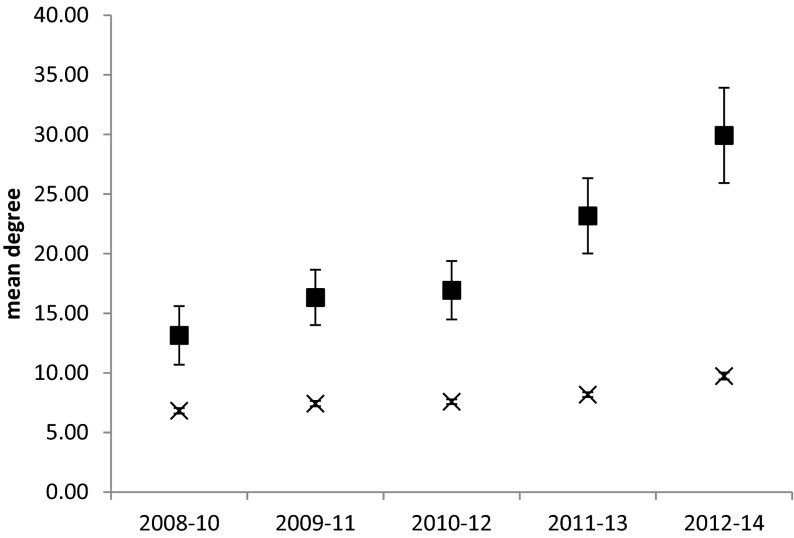
Mean (±SE) degree centrality of authors who are members of the MACSUR knowledge hub (*squares*) compared with remaining authors in the network (*crosses*). Mean degree centrality is significantly higher for MACSUR members (*F*_1,10332_ = 457.1, *p* < 0.001) and significantly different between time periods for MACSUR members (*F*_1,10332_ = 457.1, *p* < 0.001)

**Fig. 4:**
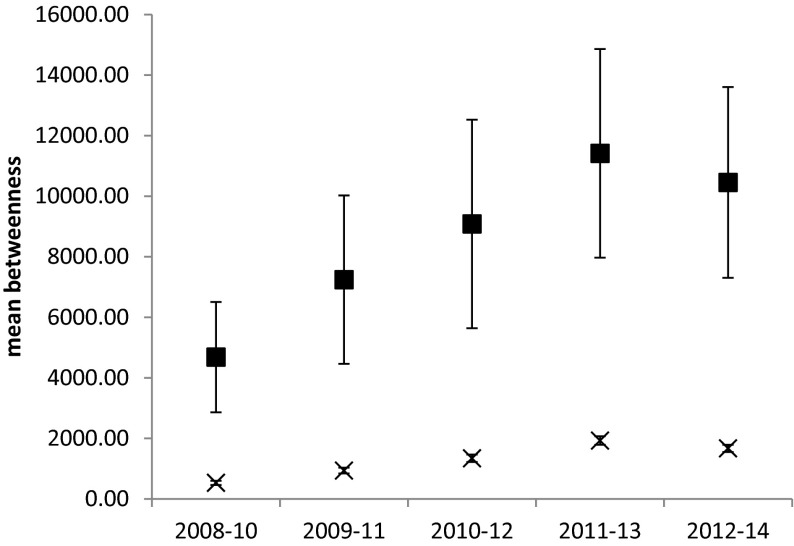
Mean (±SE) betweenness centrality of authors who are members of the MACSUR knowledge hub (*squares*) compared with remaining authors in the network (*crosses*). Mean degree centrality is significantly higher for MACSUR members (*F*_1,10332_ = 375.1, *p* < 0.001) and significantly different between time periods for MACSUR members (*F*_1,10332_ = 38.23, *p* < 0.001)

MACSUR members appeared to be better connected than other authors in the whole community network, with a significantly higher degree centrality (Fig. 3) and higher betweenness centrality (Fig. 4) throughout the study period. Degree centrality of non-members remained stable, but for MACSUR members it increased rapidly during the active phase of the knowledge hub (Fig. 3). Betweenness centrality increased more gradually throughout the period of study (Fig. 4).

## Discussion

In order to evaluate the impact of the MACSUR knowledge hub as a tool for fostering cohesion and capacity building across scientific disciplines, the development of collaboration was studied by applying network analysis to a database of co-authored peer reviewed journal papers. Evidence was found for an effect of the knowledge hub, with the degree centrality of MACSUR members showing a significant increase during the active period of the project. Network centrality of an author has been positively correlated with measures of performance (Abbasi et al. 2011; Lee 2005; Uddin et al. 2013) with authors having a larger numbers of collaborators also more likely to engage in new collaborations. The observed increase in degree centrality within the MACSUR members’ network is therefore a positive sign in terms of the impact of the knowledge hub on scientific capacity.

Initially, analysis showed the MACSUR community to be highly fragmented, but the connectedness and size of the largest network component grew throughout the study period, with a considerable shift in network structure between the third and fourth time-windows as this large component united the network. This mirrors earlier observations of network development (Liu and Xia 2015; Tomassini and Luthi 2007). The catalyst for the quick growth of such core clusters can be unclear (Liu and Xia 2015), although for the “Genetic Programming” community studied by Tomassini and Luthi (2007) the appearance of the dominant component coincided with the first general conference in the field. Newman (2001) found that authors who have neighbours in common (i.e., authors who have collaborated with the same co-authors) have a higher probability of themselves becoming collaborators, so that fast growth in network connectivity arises through a process of preferential attachment between members. Liu and Xia (2015) identified three stages in this pattern of growth in co-authorship networks: “segregation”, “chained communities” and “small world”. An initial merging of small collaborative clusters is seen within disciplinary boundaries, which then form the nuclei for linking to other relatively close disciplines. Finally, the emergence of a large single component occurs as a result of linking multiple disciplinary communities. This final stage represents much greater interdisciplinary collaboration, and convergence of research themes into a new paradigm (Liu and Xia 2015). It can be expected to lead to a densification of the network (Bettencourt et al. 2009). For the network of MACSUR members, the high degree of densification and the shift towards a single large component coinciding with the establishment of the knowledge hub, suggests that the research structure of the project may have played an instrumental part in initiating the change.

Among the early outcomes of collaboration within MACSUR were several multi-author review papers, bringing together authors from many disciplines (e.g. Ewert et al. 2014; Rötter et al. 2013; Trnka et al. 2011). The publication of these ‘position’ papers can to some extent explain the observed changes in the network, and can be seen as evidence of synergy developing between disciplines as the result of short-term project-based collaboration (Siedlok and Hibbert 2014). It remains to be seen whether continued work within MACSUR can foster longer term re-configuration of disciplines and the formation of a stable community of researchers repeatedly engaging in interdisciplinary work (Siedlok and Hibbert 2014). However, considering that the members of MACSUR are drawn from very diverse disciplines such as crop science, economics and livestock science, the observed changes are a positive sign of growing interdisciplinary collaboration within the knowledge hub.

The MACSUR knowledge hub has only been in existence for 3 years, and previous experience shows that a much longer period (up to 10 years) may be needed for the impact of enhanced collaborative opportunities to translate into dramatic increases in numbers of co-authored papers (Tomassini and Luthi 2007). Among the challenges to developing long-term stable communities of inter-disciplinary researchers are the lack of shared framing of topics and a lack of interaction between scientists (Brandt et al. 2013). EU funding policies have facilitated the development of research networks which are much more diverse and spread over a greater geographical distance than many other collaborative efforts (Gusmao 2001). Although it has often been hard to say whether this approach has promoted the formation of a genuinely cohesive European scientific community (Gusmao 2001) specific networks have shown some evidence of integration, even when participants have been drawn from a wide range of backgrounds (Janssen et al. 2006). The network studied here shows the establishment of interdisciplinary collaborations, through co-authorship links between authors from different disciplines within the MACSUR community (Fig. 2). For MACSUR, success in sustaining the observed improvements will be a further important milestone for the knowledge hub.

Although the degree centrality of MACSUR partners was observed to rise during the active period of the project, this group of researchers also began with a significantly higher degree centrality and betweenness centrality than other authors in the Climate Change and agriculture community (Fig. 4). Authors with more experience of networking and an open attitude to collaborative working may have been more motivated to join MACSUR, or their connections might have meant that they were better informed about the opportunity to take part. The high betweenness centrality of MACSUR members suggests that these authors may have a sizeable influence over the information flow in the research community (Newman 2004) and may therefore attract more new co-authors, further cementing their control over information flow through the network (Abbasi et al. 2012). In some cases these kinds of pattern may lead to the emergence of exclusive groupings of researchers centred on a few key knowledge brokers (Abbasi et al. 2012) and present barriers to entry for others; research from other domains also suggests that over-stability within collaborations can have a negative effect on performance (Turrini et al. 2010). In this context, identifying ways to widen participation in knowledge hubs and similar networking and research structures will be important to making full use of the diversity of research expertise available in the EU and beyond.

The search for papers in the field of Climate Change and agriculture beyond the MACSUR members’ network revealed a very large and active research community with a steady increase in publications apparent between 2008 and 2014. The density and connectedness of the resulting co-authorship network was very low, suggesting that this is not as yet a cohesive community. The network of all authors could be broken down into a large number of components, with a high degree of connectedness within the components and low connectedness between them. This is consistent with the experiences from other interdisciplinary fields (Fields 2015; Janssen et al. 2006; Liu and Xia 2015). The fast growth of the core component of the MACSUR members’ network was not visible in the network formed by the wider field of Climate Change and agricultural research. Instead, a continued and gradual increase both in the overall network diameter and in the size of the largest component was observed. Although this is suggestive of an increase in interdisciplinary links throughout the study period, this growth could largely be accounted for by the overall increase in author numbers.

Fostering interdisciplinary collaborations is clearly challenging, considering the diverse commitments of the potential participants. Communication within such collaborations require a degree of translation (Sundberg 2007) as all actors in the partnership do not necessarily share the same vocabulary. This becomes an increasingly important aspect when collaboration not only crosses disciplinary but also cultural and national boundaries. The strength of collaborations tend to decrease with distance, with progress hindered by language, cultural or institutional barriers (Pan et al. 2012). As has been documented in other inter-disciplinary networks (Janssen et al. 2006), the development of an integrated inter-disciplinary community can be a slow and gradual process and (particularly in relation to collaborative publications) can be hindered by a lack of high quality inter-disciplinary journals (Janssen et al. 2006). Network analysis seems to show that the formation of the MACSUR knowledge hub enabled members to overcome such barriers to some extent, with good integration between themes evident in the final network (Fig. 2). Personal compatibility can be more important than external incentives for fostering collaboration (Hara et al. 2003; Lee 2005) and it may be that the already-well connected partners of MACSUR were able to make best use of the opportunities offered by the project as a result of a pre-existing tendency to, and experience of, collaboration. Becoming aware of and getting to know potential collaborators can be a significant limiting factor in forming new working relationships (Cainelli et al. 2014; Hara et al. 2003; Tomassini and Luthi 2007) and this might also be true for identifying networking structures and opportunities that could ease the process. Once MACSUR was formed, regular meetings and focus on communication and translation within the community were used to help members overcome some of the barriers to collaboration. As communication between partners continues, the resultant building of trust and understanding across disciplinary and cultural boundaries might be expected to facilitate further growth of community cohesiveness.

Looking to the future, the lessons from MACSUR and other collaborative research initiatives should be considered and improvements incorporated into the design of new research structures. However, significant differences can be seen among different research fields in the structure of collaborative networks (Newman 2004; Tomassini and Luthi 2007) stemming from differences in culture and history. This means that a single type of organisational structure is unlikely to be effective in all the fields to which it is applied. Instead of attempting to develop a ‘one-size-fits-all’ solution to collaborative research, the literature on inter- and trans-disciplinary interactions should be mined to identify the common components of success, while maintaining flexibility in implementation to meet the needs of individual circumstances. Many researchers in the Climate Change and agriculture community did not appear to be part of the increasing cohesion observed within MACSUR. This means that, while the scientific and personal benefits of network development need to be recognised, continuing efforts are also required to ensure that research communities remain open to new inputs and perspectives from potential collaborators in the wider network.

This paper sought to describe the potential of network analysis to characterise the success of a major inter-disciplinary initiative in growing collaborative links between researchers. Previously several such analyses have been carried out within disciplinary fields (e.g. mathematics: Brunson et al. 2013; Industrial Ecology: Kim and Perez 2015); moving beyond this to the inter-disciplinary level posed methodological challenges, particularly around the definition of the boundaries of the research network. A more focused database search might have yielded a narrower range of papers, and perhaps identified a more close-knit network of Climate Change and agricultural researchers. However, it would have missed a large number of MACSUR members and would not have fully represented the interdisciplinary and broad nature of the MACSUR knowledge hub or the network from which it is drawn.

Throughout the study period, the majority of papers identified across the Climate Change and Agricultural field were co-authored by more than two authors, and by authors from different institutions. Generally, science has seen an increase in between-institute collaboration, with multi-institute partnerships the fastest growing form of authorship (Jones et al. 2008; Leydesdorff and Wagner 2008; The Royal Society 2011). Over time, geographic distance is becoming less important as a determinant of collaboration (Jones et al. 2008), though language and culture still present significant barriers to collaboration (Hoekman et al. 2010; Katz 1994; Katz and Martin 1997; Luukkonen et al. 1992). Therefore, assessments of success for projects aimed at capacity building through collaboration need to compare performance against changes in the wider background community, in order to ascertain their real added value. In this study, changes in the structure of the MACSUR partners’ network were distinct from those observed at the level of the broader Climate Change and agriculture network, indicating a positive impact of MACSUR even a relatively short time after its initiation.

## Conclusions

Specific funding initiatives, such as the FACCE JPI knowledge hub MACSUR, are aimed at increasing international and inter-disciplinary collaboration, in order to increase capacity to tackle the challenges of Climate Change. Here, a network analysis approach was taken to try to assess the success of the MACSUR initiative in forming links between researchers across disciplines, in the context of changes in the wider research network. Research in the field of Climate Change and agriculture is growing and diverse; across this inter-disciplinary field network analysis revealed a low level of cohesion, as represented by incidents of co-authorship. However, despite the previously recognised barriers to inter-disciplinary integration, the community of MACSUR members displayed an increase in network connectedness coinciding with the establishment of the knowledge hub, and exceeding the gradual increase in connectivity in the broader research field. Results indicated that the project may have attracted an already well-connected sub-set of researchers, and a future challenge for academics and funding organisations will be to involve and gain from the diversity of expertise that exists beyond already well-integrated institutes and researchers. This study was undertaken after only 3 years of the MACSUR project, while establishing lasting collaborations can be a lengthy process. In this context, the fact that network analysis could already provide evidence of success was encouraging. Maintaining this increased cohesion in the long term will require continued support for networking activities, to counter institutional and personal barriers to collaboration, and to enable scientists to work together more effectively to address complex societal challenges.
